# Effects of Al_2_O_3_, SiO_2_ nanoparticles, and g-c_3_n_4_ nanosheets on biocement production from agricultural wastes

**DOI:** 10.1038/s41598-023-29180-0

**Published:** 2023-02-15

**Authors:** Essam M. Abdelsalam, Mohamed Samer, Amira Seifelnasr, Mohamed A. Moselhy, Hatem H. A. Ibrahim, Maryam Faried, Yasser A. Attia

**Affiliations:** 1 Department of ​Laser Applications in Metrology, Photochemistry and Agriculture (LAMPA), National Institute of Laser Enhanced Sciences (NILES), Cairo University, Giza, Egypt; 2 Department of Agricultural Engineering, Faculty of Agriculture, Cairo University, Giza, Egypt; 3 Department of Microbiology, Faculty of Agriculture, Cairo University, Giza, Egypt; 4 Department of Structural Engineering, Faculty of Engineering, Cairo University, Giza, Egypt

**Keywords:** Environmental biotechnology, Nanobiotechnology, Environmental impact, Pollution remediation

## Abstract

Environmental issues are brought up concerning the production of Portland cement. As a result, biocement serves as a reliable substitute for Portland cement in green construction projects. This study created a brand-new technique to create high-quality biocement from agricultural wastes. The technique is based on nanomaterials that improve and accelerate the "Microbially Induced Calcite Precipitation (MICP)" process, which improves the quality of the biocement produced. The mixture was further mixed with the addition of 5 mg/l of graphitic carbon nitride nanosheets (g-C_3_N_4_ NSs), alumina nanoparticles (Al_2_O_3_ NPs), or silica nanoparticles (SiO_2_ NPs). The cement: sand ratio was 1:3, the ash: cement ratio was 1:9, and water: cement ratio was 1:2. Cubes molds were prepared, and then cast and compacted. Subsequent de-molding, all specimens were cured in nutrient broth-urea (NBU) media until testing at 28 days. The medium was replenished at an interval of 7 days. The results show that the addition of 5 mg/l of g-C_3_N_4_ NSs with corncob ash delivered the highest “Compressive Strength” and the highest “Flexural Strength” of biocement mortar cubes of 18 and 7.6 megapascal (MPa), respectively; and an acceptable “Water Absorption” (5.42%) compared to all other treatments. This treatment delivered a “Compressive Strength”, “Flexural Strength”, and “Water Absorption” reduction of 1.67, 1.26, and 1.21 times the control (standard Portland cement). It was concluded that adding 5 mg/l of g-C_3_N_4_ NSs to the cementitious mixture enhances its properties, where the resulting biocement is a promising substitute for conventional Portland cement. Adding nanomaterials to cement reduces its permeability to ions, increasing its strength and durability. The use of these nanomaterials can enhance the performance of concrete infrastructures. The use of nanoparticles is an effective solution to reduce the environmental impact associated with concrete production.

## Introduction

Biocement is a novel green building material made using agricultural wastes. The utilization of biocement has demonstrated environmental, economic, and technical advantages. The resulting concrete is called “green concrete”^1,2^. Biocement considerably improves mortar resistance against acid attacks. Moreover, biocement mortar has improved resistance to water permeability than Portland cement alone^3^. De Muynck et al.^4^ coined the following terms: biomineralization or biodeposition of CaCO_3_, biomortar, and bioconcrete which are made from biocement. The following biowastes can be used as feedstocks for biocement production: rice husk, rice straw, vetiver grass, corncob, sugarcane, oil-palm shell, wheat straw, flax stem, bamboo leaf, sewage sludge, microalgae, sawdust, and paper mill sludge^3–7^.

“Nanotechnology” can be defined as the study, exploitation, and use of materials between 1 and 100 nm in size called “Nanomaterials”, where 1 nm (nm) is equal to 10^−9^ m. The nanomaterials can be synthesized in the form of nanocubes, nanowires, nanorods, nanotubes, and nanoparticles (nanospheres and nanocapsules). The key characteristics of nanomaterials vary fundamentally from the original material^8^.

The retrogradation of cementitious constructions is a widespread problem since they have elevated permeability, letting water intrude, and leading to corrosion. The implementation of sealers, e.g., biocement, is a powerful means to boost concrete durability^9^. Ash of agricultural wastes can be added to replace only 6–20% of the Portland cement. The biocement strength declines upon using higher organic residues ash^1^. This hinders the expansions in the use of biocement and limits the environmental benefits of using biocement as well.

Nanomaterials are hypothesized to enhance the binding abilities among the different components of cementitious materials. Thus, using nanomaterials allows the addition of organic residues ash to replace an amount higher than 20% of the Portland cement while maintaining the strength of the produced biocement. Consequently, this positively affects the engineering properties, especially mechanical properties, of the produced mortar and concrete from biocement. Additionally, nanomaterials are hypothesized to biostimulate the bacteria and increase their activity which accelerates biomineralization leading to a rise in the amount and rate of CaCO_3_ precipitation. This ultimately leads to sealing the concrete cracks. Nanomaterials such as nano-silica (nano-SiO_2_), nano-alumina (nano-Al_2_O_3_), nano-ferric oxide (nano-Fe_2_O_3_), nano-titanium oxide (nano-TiO_2_), carbon nanotubes (CNTs), graphene, and graphene oxide can be mixed with cement-based materials^10^. Several researchers have studied the incorporation of nanomaterials into cement-based materials in recent years. The combination of cementitious composites and nanomaterials has the potential to improve the mechanical strength of the resulting concrete structures^11–15^. Nano-silica is a common nanomaterial used in cement-based composites. This material hastens cement hydration by generating calcium-silicate-hydrate (C–S–H) and dissolving tricalcium silicates (C3S)^16^. Furthermore, the nano-silica acts as a seed for C–S–H nucleation, which accelerates cement hydration^16^. The addition of nano-silica to cement-based materials can improve their durability, workability, and mechanical properties^17^. Nano-Al_2_O_3_ particles, on the other hand, can increase the compressive strength of cement-based materials^18,19^. With a dosage of 0.25% by cement weight, Al_2_O_3_ nanofibers can increase the compressive strength of cement-based materials by up to 30%^16^. Nanoparticles improve the strength and durability of concrete by stimulating the hydration reaction and filling the micropores in the cement paste structure. This decreases the porosity of concrete, improving cement mortar's strength and mechanical properties^10^.

This study aims to reinforce biocement produced from agricultural wastes using nanomaterials. The specific objectives are as follows: increasing the amount of agricultural waste ash that can be added to the Portland cement mixture to produce biocement, while maintaining the engineering properties of the produced biocement. Enhancing the binding abilities among the different cementitious materials by using nanomaterials. Biostimulation of bacterial cells to accelerate biomineralization leads to a rise in the amount and rate of CaCO_3_ precipitation that seals concrete cracks. Investigating the following engineering properties of the resultant biocement: “Compressive Strength”, “Flexural Strength”, and “Water Absorption”.

## Results

### Bacteria isolation and cultivation conditions

**Figure 1:**
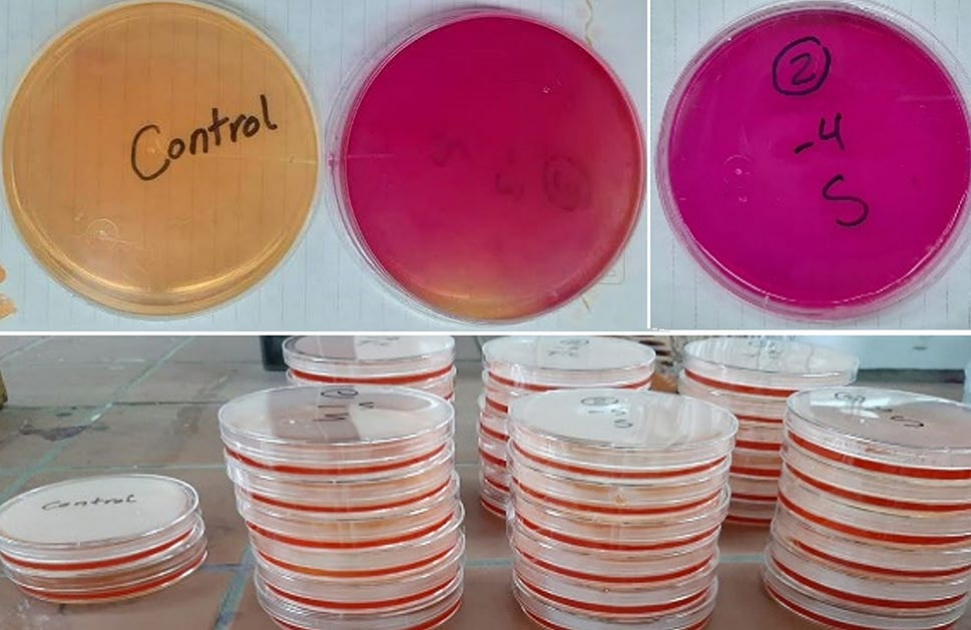
Qualitative urease activity on slants after 24 h of incubation (2% urea).

The capability of bacteria to hydrolyze urea using urease was tested on solid media, Christensen’s Urea Agar Base (UAB). Figure 1 presents the qualitative urease activity on slants after 24 h of incubation. Positive urease activity occurred in slants where it turned from yellow to pink compared to the control negative (un-inoculated medium) which did not change from yellow, and the release of ammonia odor was detected. This rise in pH makes the indicator change from yellow to pink as described by Prescott^20^ and Hammad et al.^21^. Twenty-eight isolates were acquired from the “enrichment culture technique”. Ten strains of bacteria were from the soil sample, eight strains from the wet sludge sample, and seven from the dry sludge sample.

### Selection of potent urease producer

**Figure 2:**
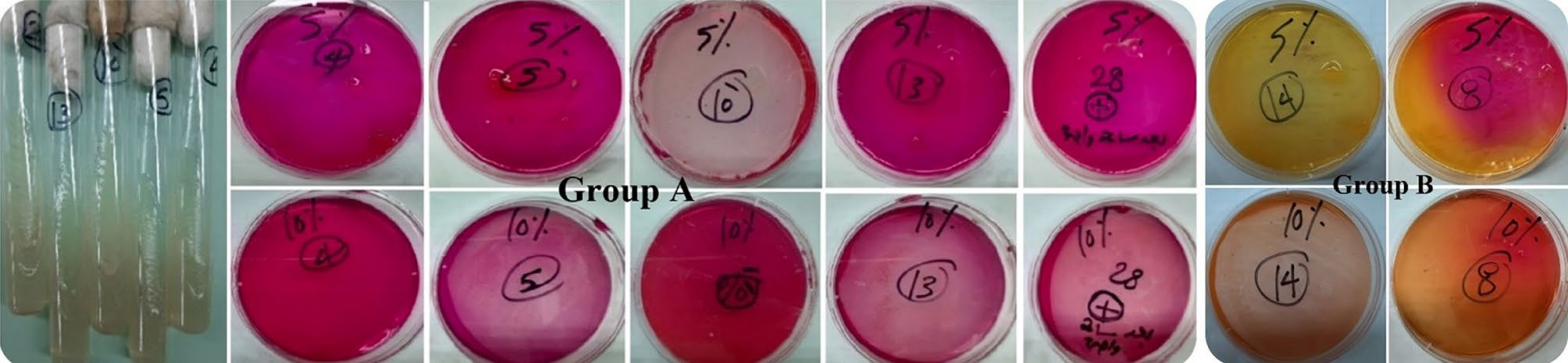
Selected strains for urease activity using Christensen’s agar (Group A: positive for urease test, Group B: ineffective at 5% and 10% urea).

“Qualitative Urease Assay” was conducted, where twenty-eight strains were chosen according to colony morphology and examined for urease activity using Christensen’s agar as described by Anitha et al.^20^. Out of the 28 strains, 5 strains (Fig. 2: Group A) were positive for the urease test and capable to make Christensen’s agar plate totally pink in 24 h at 5% and 10% of urea concentrations. This isolate was designated as numbers (4, 5, 10, 13, 28) and used for biocement production. The other ineffective strains (Fig. 2: Group B) were excluded.

### Urease activity test

“Quantitative Urease Assay” was conducted using a conductometer was preferred in this investigation since it is a precise and dependable method. When the enzyme was added to the substrate, the conductivity was measured at minutes 0, 15, 30, 45, and 75, where 5 types of bacteria were examined (Table 1).

**Table 1:** Results of urease activity test in terms of electrical conductivity (mS/m).

Strain No	Urease activity test (mS/m)			
	0 min	15 min	45 min	75 min
Strain 4	0.68 ± 0.13	0.70 ± 0.15	0.70 ± 0.15	0.69 ± 0.15
Strain 5	0.57 ± 0.03	0.59 ± 0.03	0.59 ± 0.03	0.59 ± 0.03
Strain 10	0.65 ± 0.07	0.66 ± 0.07	0.67 ± 0.06	0.66 ± 0.06
Strain 13	0.73 ± 0.03	0.74 ± 0.03	0.75 ± 0.01	0.77 ± 0.01
Strain 28	0.89 ± 0.06	0.88 ± 0.06	0.85 ± 0.08	0.85 ± 0.07

The elevated correlation coefficients indicated a powerful dependency between conductivity rise and urea hydrolysis; which made the enzyme activity which was termed by the rate of conductivity rise^23^. The results were 1.63, 3.4, 4.75, 5.88, 7.13, 8.86, 10.31, 28.7, 32.6, and 34.5 mS/m for day 1, 2, 3, 4, 5, 6, 7, 14, 21, and 28, respectively. Accordingly, an increase in the value of electrical conductivity was observed over the period of 28 days, which indicates the growth and activity of bacteria and the breakdown of urea, and thus CaCO_3_ precipitation.

### CaCO_3_ precipitation zones on agar plates

**Figure 3:**
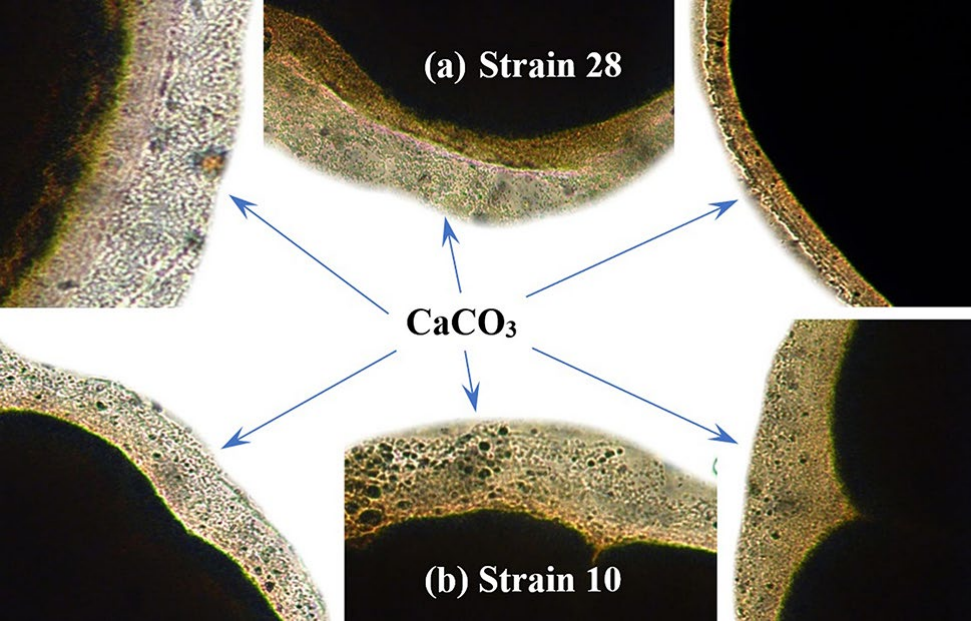
CaCO_3_ precipitation zones by microscopy (after 6 days of incubation), where (**a**) strain 28, and (**b**) strain 10.

CPA medium was implemented for CaCO_3_ precipitation^24^. A white precipitate of CaCO_3_ crystals was precipitated in agar. Figure 3 presents the CaCO_3_ precipitation after 6 days of incubation.

After inoculating the NB-U/Ca, a white powder was observed in the medium and increased through incubation. After 7 days of incubation, the CaCO_3_ precipitate was gathered and weighed. Table 2 presents the weights of the precipitate. The formation of CaCO_3_ precipitate was because of the hydrolysis of urea which resulted in producing ammonia and carbonate. Ammonia increases the pH of the media, which encourages the precipitation of CaCO_3_. Carbonate attaches calcium ions available in media resulting in the creation of CaCO_3_ crystals which were accumulated in agar and broth medium as described in the literature^24,25^

**Table 2:** The weights (g) and pH of carbonate precipitate.

Group A strains	Weight of CaCO_3_ (g)	pH
Strain 4	0.62 ± 0.05	8.76 ± 0.04
Strain 5	0.67 ± 0.05	8.84 ± 0.04
Strain 10	0.84 ± 0.07	9.09 ± 0.05
Strain 13	0.73 ± 0.06	8.88 ± 0.04
Strain 28	1.11 ± 0.09	9.43 ± 0.27

### Characterization of the prepared nanomaterials

#### Characterization of the prepared Al_2_O_3_ nanoparticles

**Figure 4:**
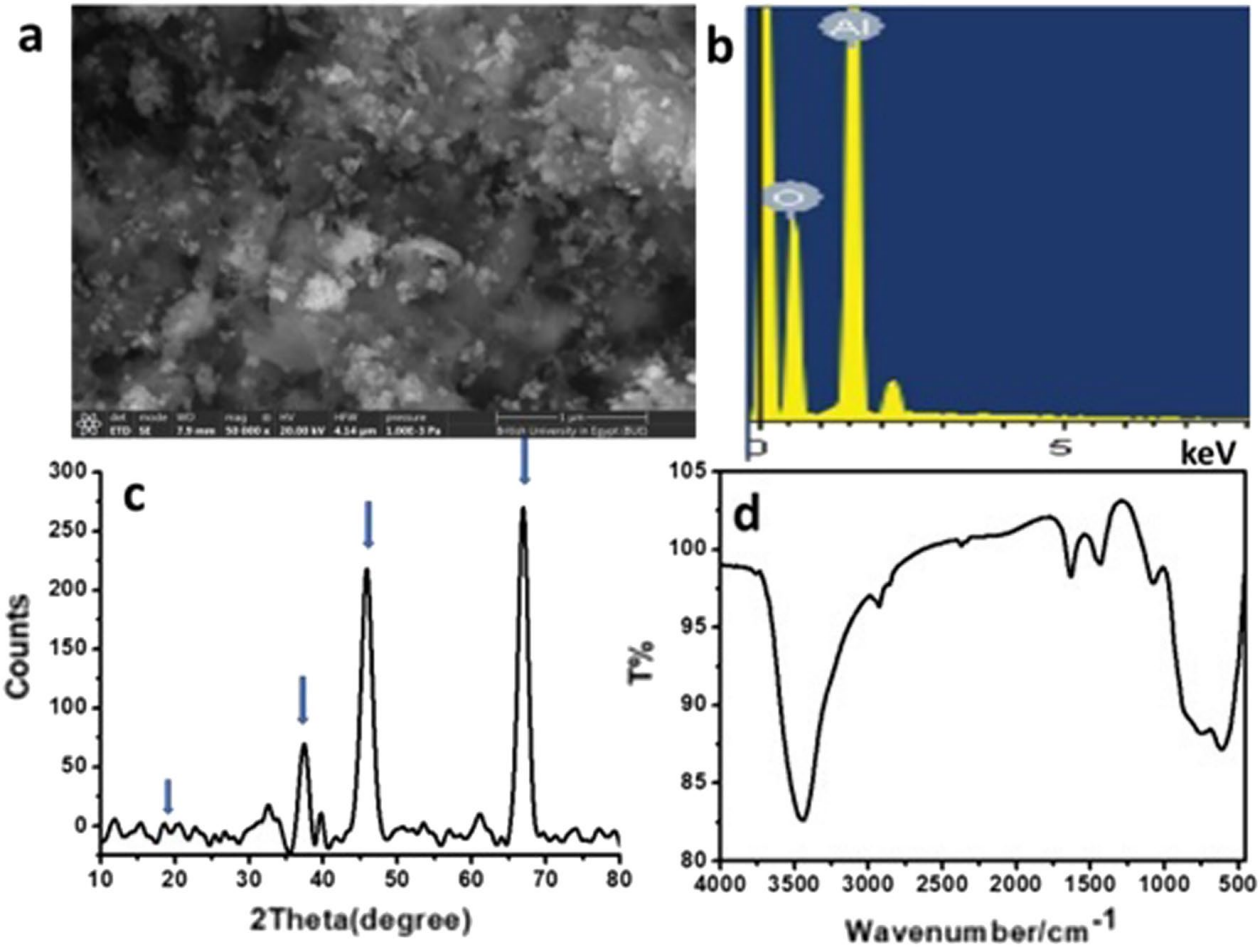
SEM image of the prepared Al_2_O_3_ NPs (**a**), EDS (**b**) shows the composition of Al_2_O_3_ NPs, XRD patterns (**c**), and FTIR spectrum (**d**), respectively.

Alumina nanoparticles were synthesized using control precipitation in an aqueous solution. The size of the prepared nano-alumina illustrated in Fig. 4a that showing the average particle size was 3 ± 0.5 nm with a spherical shape. The chemical compositions of the current aluminum oxide sample after annealing were determined using EDS and are shown in Fig. 4b. The presence of aluminum and oxygen elements with 38wt% and 62wt% without impurities is revealed by a spectrum study, which confirms the stoichiometry of aluminum oxide nanomaterials synthesized by the precipitation method. XRD was implemented for characterizing the powders of the prepared alumina nanoparticles in terms of their crystallinity degree and crystallite size. According to the standard data (JCPDS card no. 44–1482), all the peaks in Fig. 4c were well matched to the characteristic diffraction peaks of γ-Al_2_O_3_ at 19.44°, 37.59°, 45.84°, and 67.00° corresponding to (220), (311), (400), (440) planes, respectively that are in good match with boehmite (ɣ-Al_2_O_3_) phase and the particle size was assessed by Scherrer's equation, where the average of crystallite particle sizes was 3 ± 0.5 nm which agrees with the results obtained from TEM. The specific surface area is 164.182 m^2^ g^−1^, total pore volume (0.19 cc/g), and average pore size (2.315 nm). Figure 4d shows FT-IR spectra of Al_2_O_3_ nanoparticles and acid-modified Al_2_O_3_ nanoparticles, where the peak at 3455.6 cm^−1^ is the stretching vibrations peak for the hydroxyl group in Al_2_O_3_ nanoparticles. The bending vibrations peak of the hydroxyl group of Al_2_O_3_ nanoparticles appears at 1632.17 cm^−1^. Al_2_O_3_ nanoparticles have stretching vibrations peaks of Al-O at 556.16 cm^−1^ and 730 cm^−1^^26^.

#### Characterization of the prepared SiO_2_ nanoparticles

**Figure 5:**
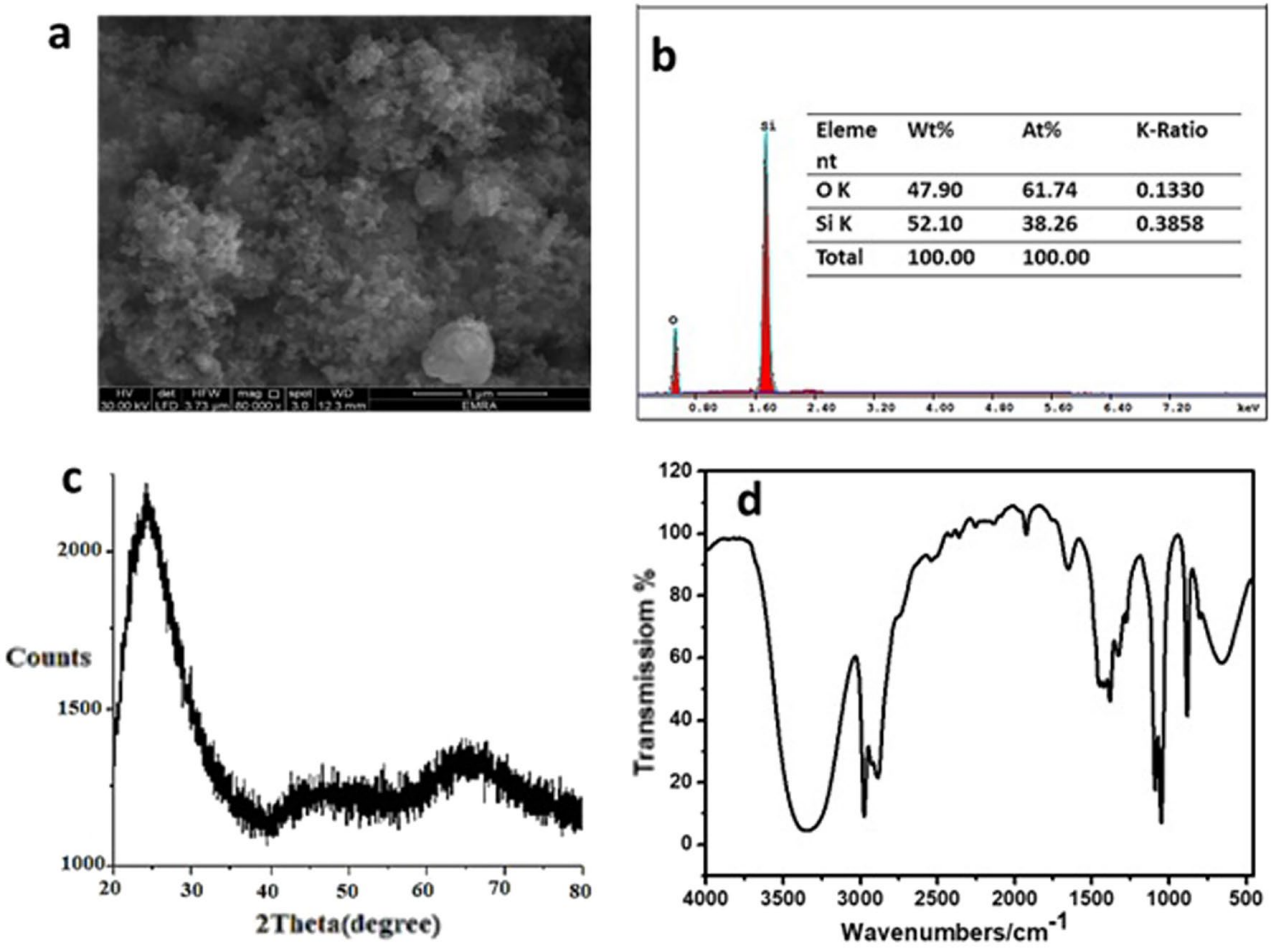
Characterization of the prepared SiO_2_ nanoparticles; SEM image (**a**), EDS (**b**), XRD (**c**), and FTIR (**d**).

SEM image of the synthesized nanosilica is presented in Fig. 5a. The spherical nanoparticles possess a satisfactory distribution, and the average particle size is about 4.0 ± 2.0 nm with an amorphous shape. EDS spectrum on a SEM (Fig. 5b) examined the chemical structure of SiO_2_ NPs. The result shows the O and Si peaks (47.9 wt% and 52.1 wt%), verifying the high purity of the sample, respectively. The specific surface area is 478.56 m^2^ g^−1^, total pore volume (0.458 cc/g), and average pore size (1.912 nm). On comparing our obtained XRD spectrum from the JCPDS Card #850,335 for SiO2, we can assure that the material formed is SiO_2_ (Quartz) nanoparticles, as the peaks reveal the formation of particles having dimensions in the nm range (Fig. 5c) and the reflection from (100), (110), (102), (111), (200) and (201) planes, respectively at 2*θ* values 20.861°, 36.550°, 39.470°, 40.296°, 42.457°, and 45.800° for the sample synthesized via sol–gel route. The XRD pattern clearly shows that the material formed has a hexagonal crystal structure. Further, FTIR spectroscopy is a useful tool to detect the binding groups in nanostructure (Fig. 5d). The peaks around 1033, 722, and 665 cm^−1^ are typical of Si–O–Si asymmetric stretching, symmetric stretching, and bending, respectively. These peaks are basic peaks indicating the silica structure, the peak around 1605 cm^−1^ for H–O–H bending vibration and a broad peak around 3425 cm^−1^ due to adsorbed water molecules which agrees with the results of Alandiyjany et al.^27^.

#### Characterization of the prepared g-C_3_N_4_ NSs

**Figure 6:**
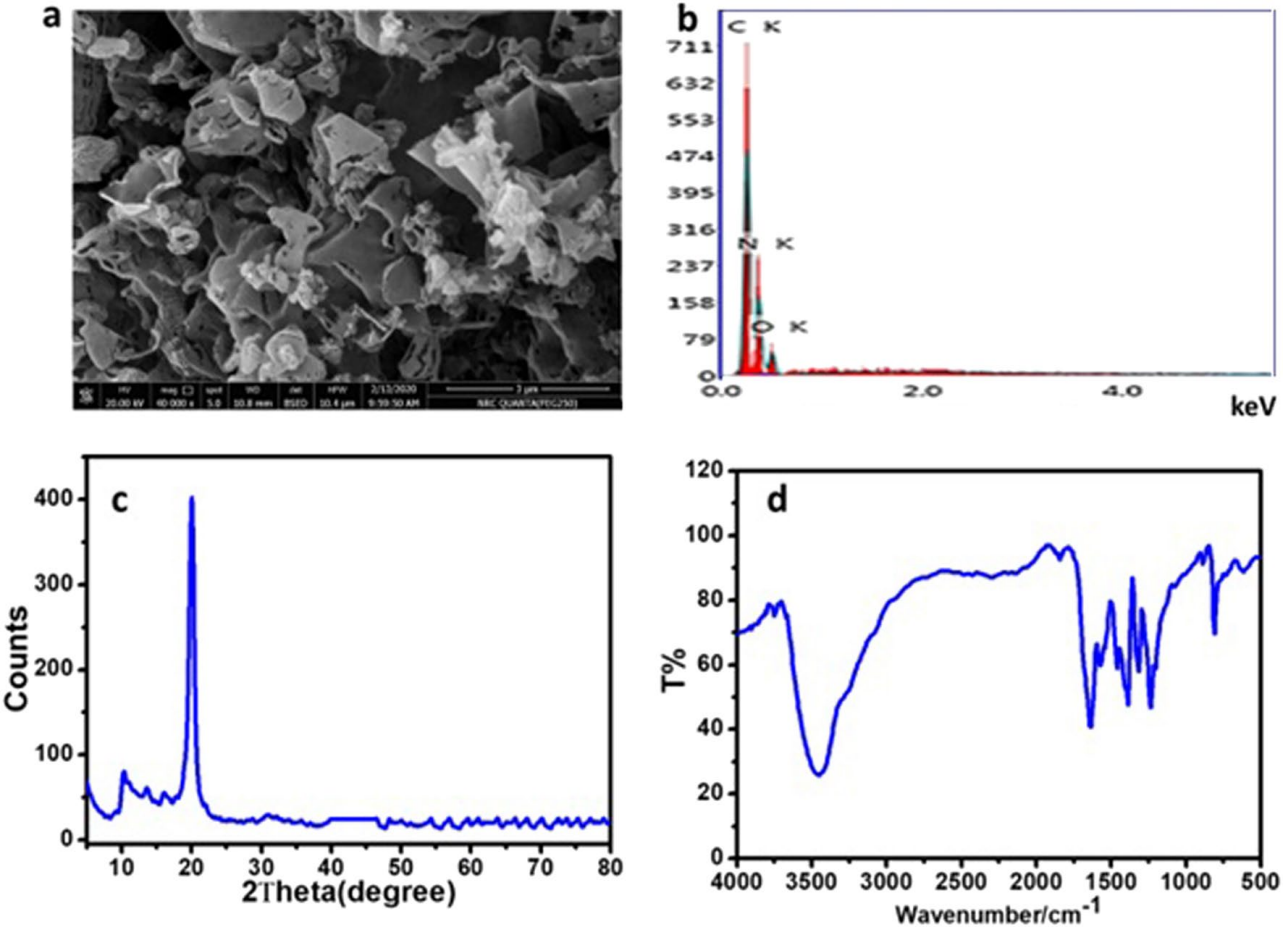
SEM image of the prepared g-C_3_O_4_ (**a**), EDS spectrum (**b**), FTIR spectrum (**c**), and XRD patterns (**d**), respectively.

The pristine g-C_3_N_4_, as typical layered and stacked structures, was observed in the sample as shown in the SEM image given in Fig. 6a which is composed of nanosheets sheets like structures and fluffier with specific surface area 99.011 m^2^ g^−1^. The EDS spectrum shows that only C, N, and O elements are present (35.27 wt% for C, 52.47 wt% for N, and 12.27 wt% for O), thus assuring the absence of any other impurities (Fig. 6b). The XRD pattern of pure-g-C_3_N_4_ nanomaterials showed that peaks at 26.73° and 13.37° can be allotted to (0 0 2) inter-layer structural packing crystal plane and (1 0 0) inter-planar stacking diffraction planes, respectively. The high peak at 26.73° demonstrates the stacking reflection of conjugated aromatic structures, uncovering a graphitic structure with an interlayer distance of 0.326 nm (Fig. 6c). The FTIR spectrum of g-C_3_N_4_, the peaks at 1145, 1213, 1393, 1587, and 1648 cm^−1^ that were ascribed to the stretching modes of CN heterocycles related to skeletal stretching vibrations of aromatic rings, whilst the peak at 810 cm^−1^ links to breathing mode of the triazine units of nanomaterials (Fig. 6d) which agrees with the results of Saeed et al.^28^.

### Compressive strength

The effects of different nanomaterials additives of alumina nanoparticles, silica nanoparticles, and graphitic carbon nitride nanosheets as well as the ash additives of rice straw, sawdust, and corncob on the “Compressive Strength” of biocement mortar cubes were evaluated. Table 3 shows the “Compressive Strength” of biocement mortar cubes after nanomaterials and ash addition compared with the control (Standard Portland Cement), where the addition of graphitic carbon nitride nanosheets as well as the addition of alumina nanoparticles and corncob ash delivered the highest “Compressive Strength” of biocement mortar cubes. Noting that, the higher the “Compressive Strength” the better the cement mortar cube.

**Table 3:** Compressive strength test (MPa) results of different nano additives to agricultural waste' bio-cement.

Agricultural wastes	Control	Nanoadditives		
		Al_2_O_3_ NPs	SiO_2_ NPs	g-C_3_N_4_ NSs
Rice Straw	17.6 ± 3.6	10.6 ± 2	10.4 ± 3.1	12.9 ± 0.7
Sawdust	14.2 ± 1.8	14.4 ± 2.9	9.8 ± 2	16.2 ± 1.3
Corncob	16.7 ± 3.6	18.1 ± 2.4	14.9 ± 3.2	18.1 ± 1.2
Portland cement	10.9 ± 2.4	‒	‒	‒

#### Flexural strength

The effects of different nanomaterials additives of alumina nanoparticles, silica nanoparticles, and graphitic carbon nitride nanosheets as well as the ash additives of rice straw, sawdust, and corncob on the flexural strength of biocement mortar cubes were evaluated. Table 4 shows the flexural strength of biocement mortar cubes after nanomaterials and ash addition compared with the control (Standard Portland Cement), where the addition of graphitic carbon nitride nanosheets and corncob ash delivered the highest flexural strength of biocement mortar cubes. Noting that, the higher the flexural strength the better the cement mortar cube.

**Table 4:** Results of the Flexural Strength Test (MPa) of different nano additives to agricultural waste' bio-cement.

Agricultural wastes	Control	Nanoadditives		
		Al_2_O_3_ NPs	SiO_2_ NPs	g-C_3_N_4_ NSs
Rice straw	6.13 ± 0.8	4.16 ± 0.9	5.26 ± 0.7	4.99 ± 0.4
Sawdust	5.77 ± 0.6	4.71 ± 0.2	3.98 ± 0.6	7.02 ± 0.6
Corncob	5.36 ± 0.3	7.02 ± 0.7	4.99 ± 0.1	7.58 ± 1.3
Portland cement	6.04 ± 0.7	‒	‒	‒

#### Water absorption test

The effects of different nanomaterials additives of alumina nanoparticles, silica nanoparticles, and graphitic carbon nitride nanosheets as well as the ash additives of rice straw, sawdust, and corncob on the “Water Absorption” of biocement mortar cubes were evaluated. Table 5 shows the “Water Absorption” of biocement mortar cubes after nanomaterials and ash addition compared with the control (Standard Portland Cement), where the biocement without any nanomaterials addition and corncob ash delivered the lowest water absorption. Besides, the addition of graphitic carbon nitride nanosheets and corncob ash delivered an acceptable “Water Absorption” of biocement mortar cubes. Noting that, the lower the “Water Absorption” the better the cement mortar cube.

**Table 5:** Results of the Water Absorption Test (%) of different nano additives to agricultural waste' bio-cement.

Agricultural wastes	Control	Nanoadditives		
		Al_2_O_3_ NPs	SiO_2_ NPs	g-C_3_N_4_ NSs
Rice straw	5.8 ± 1.6	8.9 ± 2.3	7.9 ± 3	9.6 ± 0.7
Sawdust	6.5 ± 2.3	6.8 ± 1.7	10.9 ± 0.9	5.7 ± 1.7
Corncob	4.7 ± 0.9	5.4 ± 1.7	5.1 ± 1.3	5.4 ± 0.8
Portland cement	6.9 ± 1.1	‒	‒	‒

#### Crack remediation test

**Figure 7:**
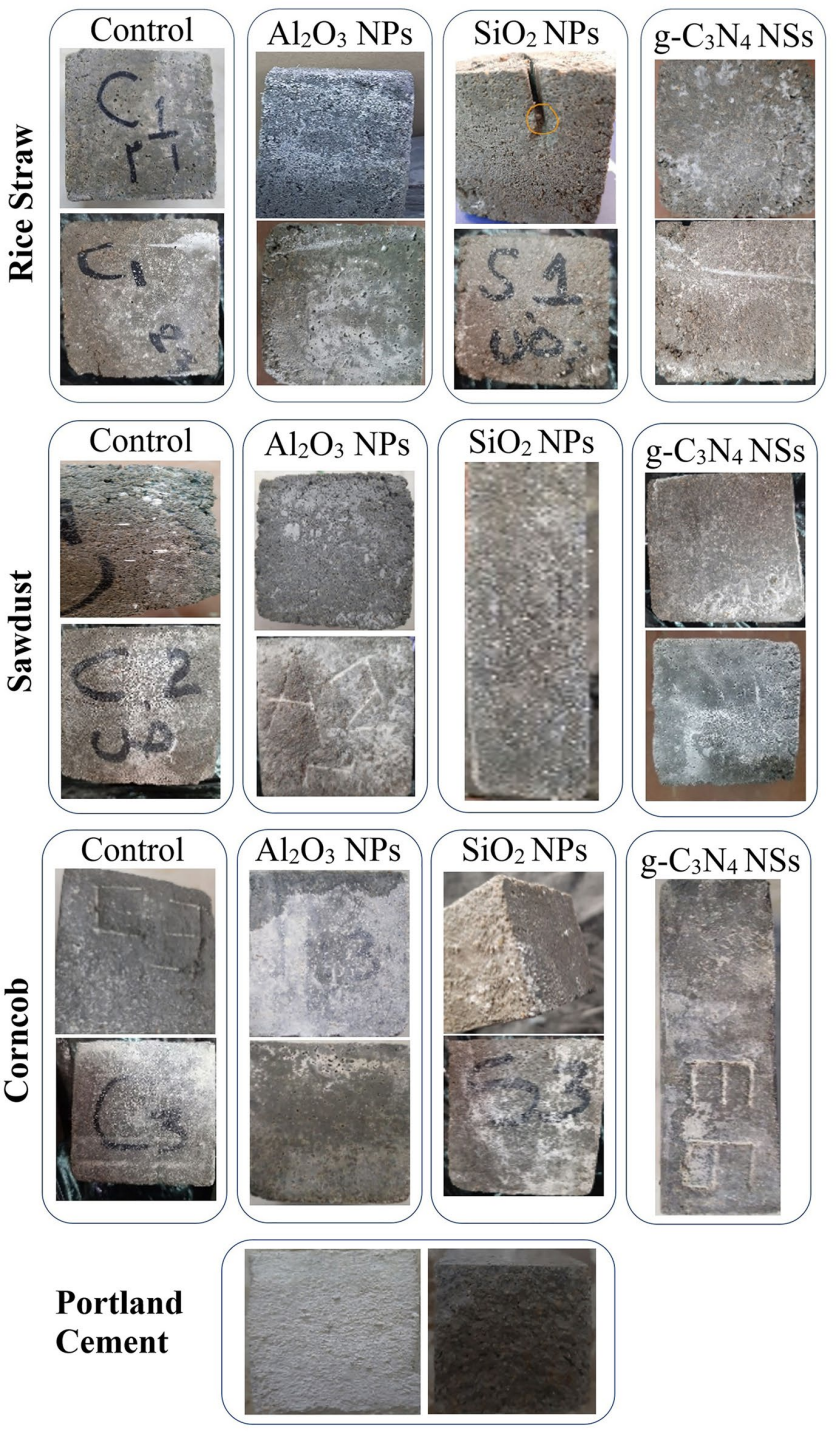
Results of the Crack Remediation Test after 28 days.

It was observed that the cracks closed gradually by calcium carbonate during the treatment period, because of the continuous growth and activity of bacteria present inside the biocement samples compared to the standard sample. The results of the test were taken regularly every 7 days over the course of treatment (28 days). It was observed that the cracks closed, and the forms of calcium carbonate precipitation changed as follows: (1) After 7 days, the formation of a solid sheet of calcium carbonate was observed on the surface of the media submerged by the cement cubes, but no change was observed on the cracks inside the samples during this period. (2) After 14 days, calcium carbonate was observed in the form of crystals on the surface of the cube and inside some cracks ranging in diameter from 1.5 to 2 ml, in addition to closing some small pores on the surface of the cube. (3) After 21 days, it was observed the formation of a white precipitate (powder) of calcium carbonate on the surface of the samples and the bottom of the vessel in which the cubes were submerged, and the relatively large cracks that ranged between 1 and 2 ml were closed. In addition to the formation of calcium carbonate crystals inside the cracks is more solid, the size and hardness of sand grains. (4) After 28 days, it was observed that calcium carbonate precipitated in the form of a chain or long solid threads emerging from the pores on the surface of the cubes, these threads were about 3 mm long and 0.5–1 mm thick, and many solid calcium carbonate crystals were formed. In addition, incisions as large as 3 mm can be closed. It was observed that some cracks that were engraved on the cube were closed. It was also observed that calcium carbonate began to form inside the deep cracks that were made by the copper piece. Figure 7 shows the results of the Crack Remediation Test after 28 days. The white precipitate is the calcite as a carbonate mineral which is the stable form of CaCO_3_.

#### Weights and densities of samples

The weights of mortar cubes ranged from 760 to 780 g, and the densities of mortar cubes ranged from 2.21 to 2.27 g/cm^3^. Table 6 shows the densities of mortar cubes after casting and demolding. Besides, Table 7 presents the densities of mortar cubes after 28 days of curing.

**Table 6:** The densities (g/cm^3^) of mortar cubes after casting and demolding.

Agricultural wastes	Control	Nanoadditives		
		Al_2_O_3_ NPs	SiO_2_ NPs	g-C_3_N_4_ NSs
Rice straw	2.1 ± 0.08	1.9 ± 0.06	2.1 ± 0.05	1.97 ± 0.06
Sawdust	2.1 ± 0.05	2.1 ± 0.05	1.98 ± 0.03	2.1 ± 0.05
Corncob	2.1 ± 0.03	2.2 ± 0.05	2.1 ± 0.03	2.1 ± 0.02
Portland cement	2.3 ± 0.03	‒	‒	‒

**Table 7:** The densities (g/cm^3^) of mortar cubes after 28 days of curing.

		Nanoadditives		
Agricultural wastes	Control	Al_2_O_3_ NPs	SiO_2_ NPs	g-C_3_N_4_ NSs
Rice straw	2.14 ± 0.07	2.04 ± 0.09	2.14 ± 0.03	2.06 ± 0.06
Sawdust	2.16 ± 0.04	2.15 ± 0.06	2.07 ± 0.01	2.16 ± 0.06
Corncob	2.17 ± 0.03	2.2 ± 0.04	2.19 ± 0.07	2.17 ± 0.06
Portland cement	2.35 ± 0.03	‒	‒	‒

#### Calcite content

**Figure 8:**
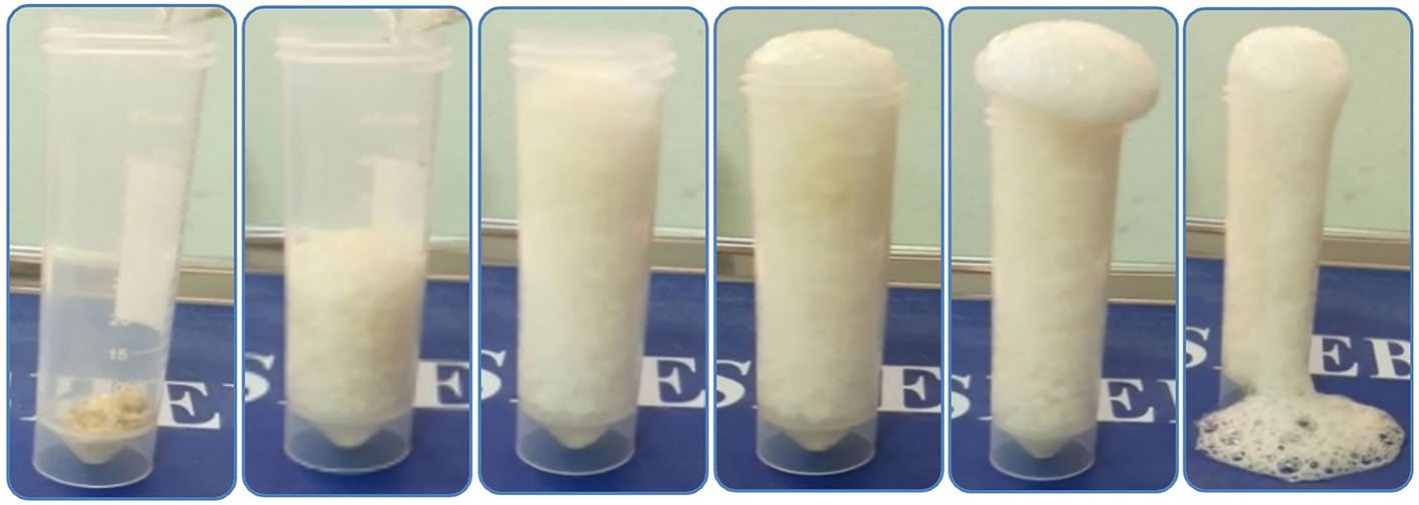
Eruption and CO_2_ release after adding 2 M of HCl to the precipitate which indicates the formation of CaCO_3_.

Upon hydrochloric acid solution addition, it reacted with calcium carbonate precipitate and, therefore; an eruption was observed, and carbon dioxide gas was released as shown in Fig. 8.

## Discussion

The world produces millions of tons of agricultural waste every year around the world which demands a profound solution to not only get rid of these wastes in an eco-friendly method but also to convert these wastes into a high-value bioproduct, this will be positively reflected in the bioeconomy. This research represents one of the unique solutions to many problems facing the world these days. In this approach, agricultural wastes are being used as a feedstock for biocement production with the addition of nanomaterials to reinforce the mortar as well as to biostimulate the bacterial cells to increase calcium carbonate precipitation to seal common concrete cracks. According to the results of this investigation, adding ash of agricultural wastes along with nanomaterials to the cementitious mixture is an easy and affordable technique with a positive outcome.

Biocement production using agricultural wastes is an environmental-friendly method and will encourage to scale-up of the process to an industrial scale to generate clean cementitious materials using an ecofriendly technique and solve the agricultural wastes issue as well. Besides, biocement is a valuable technique to mitigate the CO_2_ released through cement production. Biocement decreases clinker and energy consumption as well as CO_2_ emissions, where the raw feedstocks for biocement production are renewable biomass. In this study, it was found that the final biocement mortar has an improved performance, which agrees with the statements of Hosseini et al.^1^. Besides, it was found that biocement mortar has improved endurance to water permeability than mortar from Portland cement solely, which agrees with Adesanya and Raheem^3^.

In this study, biocement was produced as a blend of bio-silica, found within the ash of agricultural wastes, with Portland cement, where the ash production process was combined with energy generation to concurrently gain energy and ash since pyrolysis has been applied, which agrees with Hosseini et al.^1^ except that they used combustion but not pyrolysis as the present study. On the other hand, in this study biocement was produced as a mixture of bacterial culture and biomass which was produced from cheap raw materials (agricultural wastes) with inorganic chemicals (sand, aggregate, and standard Portland cement), where this agrees with production process applied by Jian et al.^19^. Furthermore, the ash of agricultural wastes was used as a replacement for limestone, sand, and iron slag used to produce biocement, which agrees with the concept of Yen et al.^7^. An important key issue is that the bacteria used to produce the biocement were found to precipitate calcite that seals cracks usually found in common concrete and mortar, which agrees with Yen et al.^7^. Moreover, these bacteria which were added to the mixture of biocement improved their compressive and flexural strength as well as their ability to remediate cracks, where MICP was implemented to remediate the cracks, which agrees with the statements of De Muynck et al.^4^. As a result, bacteria were found to be self-healing for the reduction of concrete permeability upon crack formation. Biocement can be used for crack healing in concrete constructions through MICP which has several functions in the remediation and restoration of construction materials (concrete and mortar) which agrees with Achal et al.^30^.

A key issue is the addition of different nanomaterials, where it was found that the specific surface area of silica nanoparticles is approximately 4.8 times the specific surface area of the graphitic carbon nitride nanosheets. Besides, it was found that the specific surface area of alumina nanoparticles is approximately 1.66 times the specific surface area of the graphitic carbon nitride nanosheets. Although the graphitic carbon nitride nanosheets have the lowest specific surface area, they unexpectedly delivered the best biocement mortar properties in terms of compressive strength and flexural strength which were the highest among all other nanoadditives of silica and alumina nanoparticles. These results were peculiar considering the specific surface area. On the other hand, the size of silica nanoparticles and alumina nanoparticles were similar to some extent (3–4 nm). However, the interlayer distance of the structure of the graphitic carbon nitride nanosheets was 0.326 nm which allowed these nanomaterials to deliver the best results. It was expected that the higher the surface area of the nanomaterials the higher the compressive and flexural strengths. This occurred when the silica and alumina nanoparticles were compared, where the silica nanoparticles delivered higher compressive and flexural strengths compared to alumina nanoparticles since the silica nanoparticles have a higher surface area than the alumina nanoparticles. However, this did not occur when both were compared with the graphitic carbon nitride nanosheets which have the lowest surface area and unexpectedly delivered the highest compressive and flexural strength compared to silica and alumina nanoparticles. These unexpected results were attributed to the interlayer distance of the structure of the graphitic carbon nitride nanosheets.

In this study, it was found that the biostimulation of microorganisms using nanomaterials increases their efficiency. Particularly, the nanomaterials biostimulate the activity and the bioresponse of the cells, which agrees with the statements of Saeed et al.^28^. Future research will focus on biocement production from agricultural wastes using laser photoactivated nanomaterials, where this photoactivation increases the activity of nanomaterials as described by Attia et al.^31^.

## Conclusions

In general, adding nanomaterials and ash from agricultural wastes to cementitious mixtures increases their qualities, particularly their compressive strength, flexural strength, and water absorption. As opposed to normal Portland cement, this method consumes less energy and emits fewer greenhouse gases, which are responsible for global warming and subsequent climate change. Specifically, it can be concluded that:

1. Adding 5 mg/l of g-C_3_N_4_ NSs to the cementitious mixture delivers higher compressive strength, the higher flexural strength of biocement mortar cubes, and a higher water absorption reduction of 1.67, 1.26, and 1.21 times the standard Portland cement, respectively.
2. Adding 5 mg/l of g-C_3_N_4_ NSs to the cementitious mixture enhances its properties, where the resulting biocement is a promising environmentally friendly substitute for conventional Portland cement.

The nanomaterials could decrease the cement porosity, generating a denser interfacial transition zone. In addition, nanomaterials reinforced cement can allow the construction of high-strength concrete structures with greater durability, which will decrease the maintenance requirements or early replacement.

## Methods

### Experimental setup

#### Bacteria isolation and cultivation conditions

In this study, three samples were used (soil, wet sludge, and dry sludge samples) to isolate strains able to produce urease. Christensen’s agar media were utilized. The media pH was altered to 6.8 ± 0.2. The isolates were splashed on Christensen’s agar slants and incubated at 37 °C for 48 h. Urease-producing isolates were identified by variations in media color from yellow to pink, as described by Anitha et al.^22^. Afterward, several tests were carried out to obtain the best bacterial type capable of producing urease and precipitating calcium carbonate and bearing the highest concentration of urea to be added to the biocement.

After isolating the bacteria and conducting tests and selecting the best bacterial type, it is grown to be added to the biocement mortar, it is *Staphylococcus arlettae*. Further identification of the isolate was performed using 16SrRNA gene sequencing. The DNA was isolated, and the analysis of DNA sequences was performed by using the Blastx software (BLAST). To select a potent urease producer, strains with a high level of urease activity were screened. The bacterial isolates were examined for their growth capability as described by Anitha et al.^22^ and Elmanama et al.^32^. After testing the ability of bacteria to tolerate the highest concentration of urea, the species capable of tolerating the highest concentration were selected, and some other experiments were conducted such as the Urease Activity Test, CaCO_3_ precipitation in agar plate state, and CaCO_3_ precipitation in broth state and pH. The isolated bacterial strain was further handled as described by Hammad et al.^21^.

### Agricultural wastes

**Figure 9:**
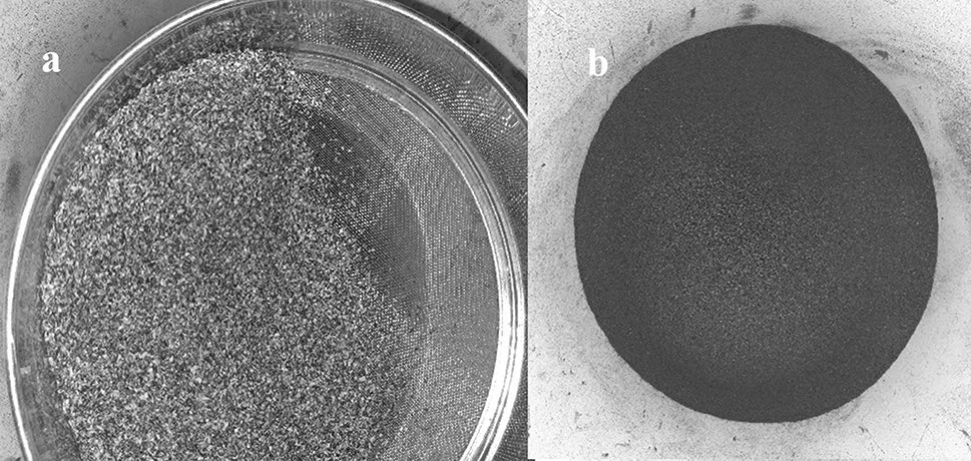
Sieving (**a**) and resulting sieved ash (**b**) at a diameter < 0.5 mm.

The following agricultural wastes were used in this study as bio-silica sources and feedstocks for biocement production: Rice straw, Sawdust of forest wastes, and Corn stover, especially corncob. Where, 20 kg of rice straw, 75 kg of sawdust, and 54 kg of corn stalks were gathered. Each residue was placed separately in the pyrolysis oven at 500–700 degrees for 5 h. The weight of the waste after pyrolysis was as follows: rice straw 2.0 kg, sawdust 2.65 kg, and corncob 2.5 kg. The produced ash was sieved using a sieve with a diameter of less than 0.5 mm, to remove the impurities and sand (Fig. 9).

### Preparation of mortar cubes

Mortar consists of cement, sand, and fine aggregates such as fly ash. Water activates the cement, which is the component responsible for binding the mixture of components together to make one hard object. In the following preparation process, agricultural wastes containing bio-silica were pyrolyzed and used as a substitute for fly ash. Pyrolysis was applied to avoid the negative environmental impacts of residue burning.

**Figure 10:**
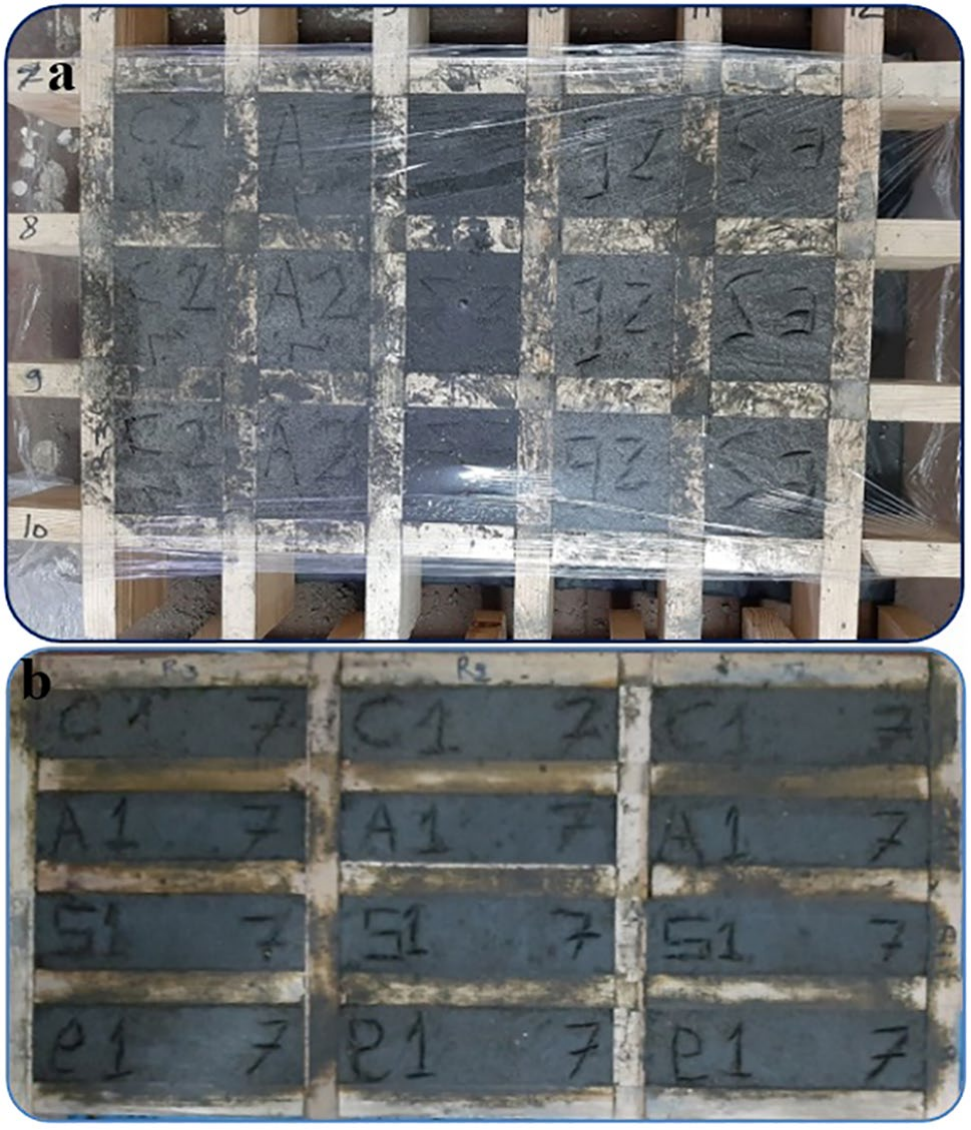
Cube wood molds containing the mortar (**a**), Prisms (**b**); Cement: sand = 1:3, Ash/Cement = 0.1, Bacterial culture/cement = 0.5.

This preparation process was performed as described in the literature^6,30,33^, but with some amendments. The preparation steps were mixing, casting, and curing. The first stage was the dry mixing of all elements (cement, sand, and agricultural waste ash) using a mortar mixer for 2 min. The second stage is casting of the treated specimens with bacteria, nutrient broth with bacterial culture (OD_600_ 0.5) with 2% urea (w/v) and 25 mM CaCl_2_ solution (w/v) were utilized instead of water and were then poured to the dry mixture, where the mixture was further blended at 140 rpm for 2 min to become homogenous. When the mixture was homogenous, the nanomaterials were slowly added, and the mixture was further mixed. Ordinary Portland cement conforming to international standards was used and blended with sand and pyrolyzed agricultural wastes. The cement: sand ratio was 1:3, the ash: cement ratio was 1:9, and the water: cement ratio was 1:2. Cement, sand, and residue ash were then blended with water and grown culture of bacterial isolate correspondence to OD_600_ 0.5. Ash of rice straw, sawdust, and corncob was added individually, and then nanomaterials were individually supplemented to the mixture which was further blended. Cube molds of dimensions 70 mm × 70 mm × 70 mm were manufactured using wood for the “Compressive Strength Test” and “Water Absorption Test”. Prism molds of dimensions 40 mm × 40 mm × 160 mm were prepared for “Flexural Strength Test”. The fresh mix was immediately transferred to wood molds (Fig. 10a, b). After casting, all specimens remained in the wood molds and were maintained at room temperature of 27 ± 2 °C for 24 h. In the third stage, the specimens were demolded and cured in nutrient broth-urea (NBU) medium at room temperature till the testing age (days) as described by Joshi et al. (2018). Media were replenished at a regular interval of 7 days. Control samples without nanomaterials, standard Portland cement without bacteria and ash, were made similarly. Three systems and two curing schemes were implemented in this investigation as specified in Table 8.

**Table 8:** Specifications of the specimens.

Specimen	Composition	Mechanism of curing
Standard	Cement: sand = 1:3 Water/cement = 0.5	Water curing for 28 days
Control (C) = Standard + Bacteria + Rice straw ash Or sawdust ash Or corncob ash	Cement: sand = 1:3 Ash/cement = 0.1 Bacterial culture/cement = 0.5	*NBU medium with urea and CaCl_2 _curing for 28 days
Nano = Control + (Al_2_O_3_ NPs) Or (SiO_2_ NPs) Or (g-C_3_N_4_ NSs)	Cement: sand = 1:3 Ash/cement = 0.1 Bacterial culture/cement = 0.5 Nanomaterials = 5 mg/kg mortar	NBU medium with urea and CaCl_2 _curing for 28 days

*NBU: nutrient broth-urea.

### Nanomaterials preparation

Two groups of nanomaterials were used in this study for two different purposes. The first group consists of nanomaterials for biocement reinforcement which are silica nanoparticles (SiO_2_ NPs) and alumina nanoparticles (Al_2_O_3_ NPs). The second group consists of nanomaterials for bacteria biostimulation which are graphitic carbon nitride nanosheets (g-C_3_N_4_ NSs).

### Alumina nanoparticles (Al_2_O_3_ NPs)

This preparation method was described by Elsayed et al.^26^. To prepare Al_2_O_3_ nanoparticles firstly, (0.066 M) aluminum nitrate solution and (0.125 M) ammonium carbonate solution was prepared, 400 ml of deionized water in a round flask with continuous stirring was taken and two previous solutions were added dropwise to precipitate Al(OH)_3_, NaOH and HNO_3_ were used to adjust pH around 8. The precipitate was heated at 70 °C for 3 h and then filtered and dispersed again in deionized water. Finally, the precipitate was filtered and rinsed with ethanol and acetone many times then dried at 30 °C. Finally, calcined the precipitate at 550 °C for 5 h with a 2 °C/min heat rate.

### Silica nanoparticles (SiO_2_ NPs)

Silica nanoparticles were prepared as described by Alandiyjany et al.^27^. Silica (silicon dioxide; SiO_2_) was extracted from rice hulls according to Battegazzore et al.^34^. Silica (silicon dioxide; SiO_2_) was extracted from rice hulls and refluxed with concentrated hydrochloric acid for 4 h with constant stirring at room temperature (20–22 °C) then washed with distilled water for acidity removal. In the next step, previously prepared silica was solved in sodium hydroxide, and to achieve pH = 8, sulfuric acid was added. Deposited silica (gel form) was rinsed several times with warm distilled water to get rid of alkalinity. To dry the formed gel, oven-dried at 55 °C for 48 h was used. To get silica nanoparticles, the solids were crushed to powder in a rapid mill^35^.

### Graphitic carbon nitride nanosheets (g-C_3_N_4_ NSs)

This preparation method was described by Saeed et al.^28^, where graphitic carbon nitride nanosheets were prepared using “Thermal Polymerization”. Specifically, 10 g of urea (> 97% Sigma-Aldrich) was ground in a mortar and then dissolved in 15 mL water (Millipore, ultrapure). The solution pH was modified to 4–5.5, thereafter it was dehydrated at 80 °C for 12 h and moved to an alumina crucible having a cover. The obtained material was then overheated up to 580 °C using a muffle furnace at 5 K min^−1^ and stayed at 580 °C for 3 h.

The morphology of the prepared nanoparticles was determined using SEM, FTIR Spectroscopy, EDS, and XRD.

### Compressive strength

**Figure 11:**
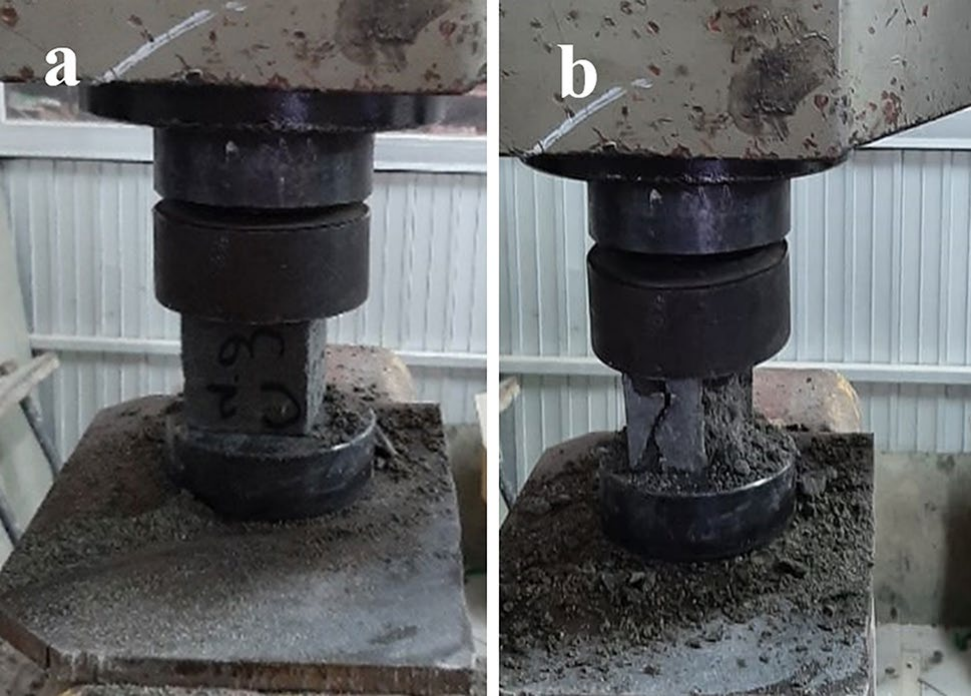
Compressing machine: (**a**) before testing and (**b**) after testing.

“Compressive Strength” is the resistance of a material to cracking under pressure. This test was conducted as described in the literature^33,36,37^ to verify the “Compressive Strength” of mortar cubes. This test was accomplished after 28 days of de-molding and hardening using an automatic compression testing machine (UH-500kNIR). The specimen was placed into the machine and gradually loaded until it cracked (Fig. 11). The final load was measured. The values are in megapascal (MPa).

### Crack area

The samples were sliced into fragments of 5 mm in thickness using a diamond saw and completely dried then SEM images were prepared. SEM analysis was conducted using gold-coated samples under a low vacuum with low N_2_ pressure at 10–20 kV of acceleration voltage before analysis by SEM. The SEM images of the specimens were employed to examine the shapes and the distribution of CaCO_3_ crystals. For the confirmation of the precipitation of calcium carbonate in bio mortar IR test was performed.

### Water absorption test

The “Water Absorption Test” was performed as described by ASTM C642-97^38,39^ to verify the rise in resistance in relation to water infiltration in mortar. The cube molds were made both with and without bacteria, agricultural waste ash, and nanomaterials. The specimens were cured for 28 days in both water and urea-CaCl_2_ solution. During subsequent curing, the surfaces of the samples were dried, and their saturated masses were determined after immersion. The specimens were then dried in an oven at 110 °C for 24 h and weighed again^40^. “Water Absorption” of the specimens was determined by the equation:

1 $${\text{Water}} \, {\text{absorption}} \, \left( \% \right) \, = \frac{W1 - W2}{{W2}}*100$$

where W1 is the mass of the sample after immersion with a dry surface and W2 is the mass of the oven-dried sample in air, all reported in g.

### Crack remediation test

**Figure 12:**
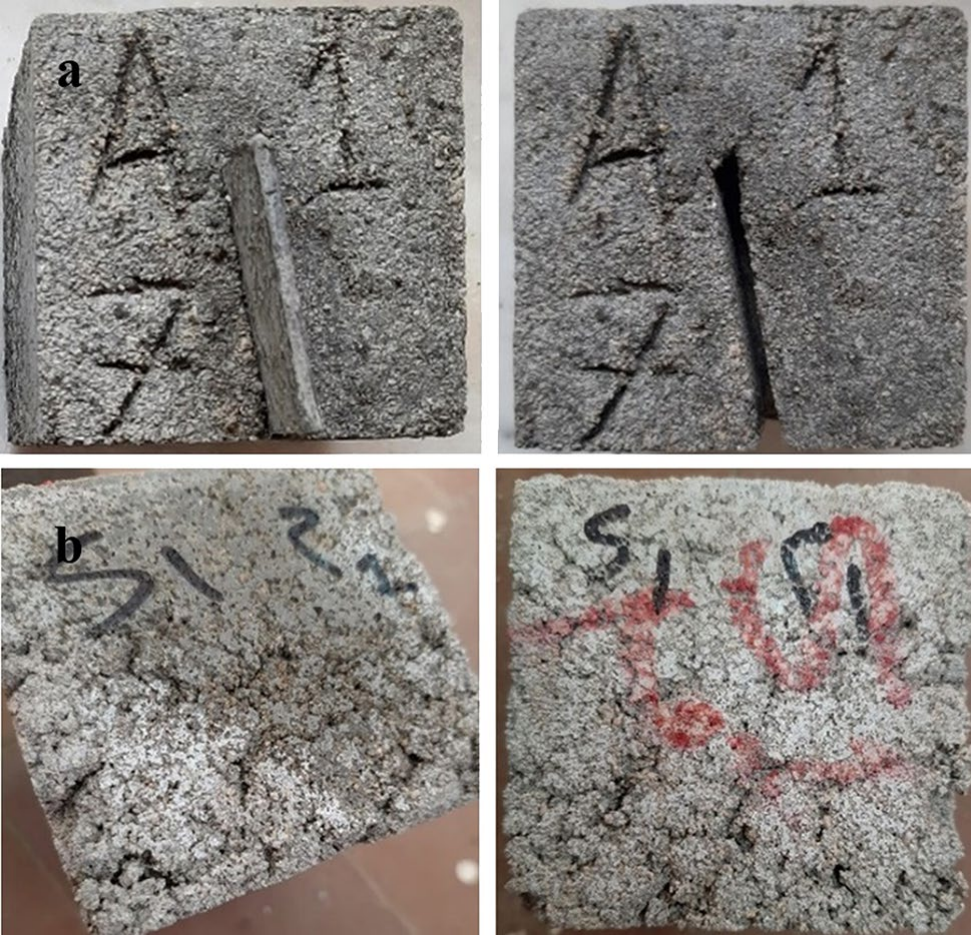
Cracks by a plate of steel (**a**), Natural cracks (**b**).

This Crack Remediation Test investigates self-healing properties. The cubes were supplied with a cut using two methods. The first method: cracks were initiated in the beam specimens by initiating a thin copperplate of thickness 3 mm up to a depth of 10 mm in concrete. The plates were detached before the final setting of concrete such that a crack was clearly visible in the beam specimens (Fig. 12a). The specimens were detached from molds after 24 h and cured in water/media including urea and CaCl_2_ for 28 days. The second method: during the preparation and pouring of the mortar cubes, the cubes were not tamped well when pouring into the molds to allow the samples to have some natural cracks that simulate the cracks that will occur in the future in the mortar or concrete, and the width of the cracks, in this case, was up to 3 mm (Fig. 12b). Photographs were taken to visualize the cracks. In a 7-day interval, the beam specimens were detached from water and the cracks were examined for the existence of white precipitate and for cracks healing. The medium was restored after an interval of 7 days. The deposition of CaCO_3_ was visually observed regularly. By end of exposure, the cubes were tested for compression, and they were also examined by SEM. Additionally, the bacterial self-healing was observed by the naked eye inducing a crack of 0.2—3 mm in size in the specimens. The healing process was observed for 28 days as described by Priya et al.^41^.

### Urease activity test

The conductivity method for urease activity assay was implemented. To conduct the enzyme assay, 1.0 ml of bacterial culture (NB-U) was poured into 9.0 ml of 1.11 M urea solution as described by Harkes et al.^42^. Ultimate conductivity was measured after 5 min of incubation at 20 ºC by an electric conductivity meter (EC Meter (. Urease activity was measured by the conductivity increase rate (mS/m) as described by Hammad et al.^21^. The urease activity of the media immersed in biocement cubes was also calculated during the treatment period for 28 days and compared to the control sample immersed in water.

### CaCO_3_ precipitation in Agar plate state

Calcite precipitation agar (CPA) is a solid medium for screening bacterial precipitation of calcium carbonate by ureolysis^24^. For CaCO_3_ precipitate screening, 20 μl of broth culture was inoculated in the plate center, and then incubated at 30 °C for 6 days. Triplicates were investigated. The plates were examined frequently to quantify the CaCO_3_ precipitate. The precipitation zone was photographed, and its diameter was determined as described by Hammad et al.^21^.

### CaCO_3_ precipitation in broth state and pH

To measure the precipitated CaCO_3_, the nutrient broth was added with 2% urea and CaCl_2_ (NB-U/Ca) and were used, where 30 ml of NB-U/Ca were inoculated with 2% then incubated under shaking condition with 130 rpm at 30ºC for 7 days. Triplicates were investigated. CaCO_3_ precipitate was filtered using filter paper (Whatman filter paper), which was dried in an oven at 60 °C for 3 h, then weighed as described by Hammad et al.^21^. CaCO_3_ precipitate weight (W_c_) was calculated using the formula:

2 $${\text{W}}_{{\text{c}}} {\text{ = W}}_{{2}} {\text{{-} W}}_{{1}}$$

where W_2_ is the weight of filter paper containing precipitate; and W_1_ is the weight of empty filter paper.

The degree of pH was also measured as an indicator of increased alkalinity because of urea decomposition, where it is a direct relationship.

### Calcite content

This test is performed to find out whether carbonates are present in a sample. As calcium carbonate increases the strength and hardness of the cement and fills the tiny pores of fractures in the concrete, it is often intended to be used as an indicator of the approximate carbonate content to confirm that the precipitate type created by bacteria is calcium carbonate. Calcite content was assessed using the gravimetric analysis of acidified samples in two ways. First, 10 g of the powder sample was implemented after oven-drying at 105 °C for 24 h. Later 2 M of HCl was poured onto the prepared powdered, where CO_2_ was released because of the reaction between calcite and HCl. The residue was gathered and oven-dried again, and the weight loss was determined before and after acid rinses, as described by Mahawish et al.^43^, the results were used to determine the percentage of calcite content in the specimen as described by Umar et al.^44^:

3 $${\text{CaCO}}_{{3}} + {\text{ 2 HCl }} \to {\text{ CaCl}}_{{2}} + {\text{ H}}_{{2}} {\text{O }} + {\text{ CO}}_{{2}}$$

### Flexural strength

**Figure 13:**
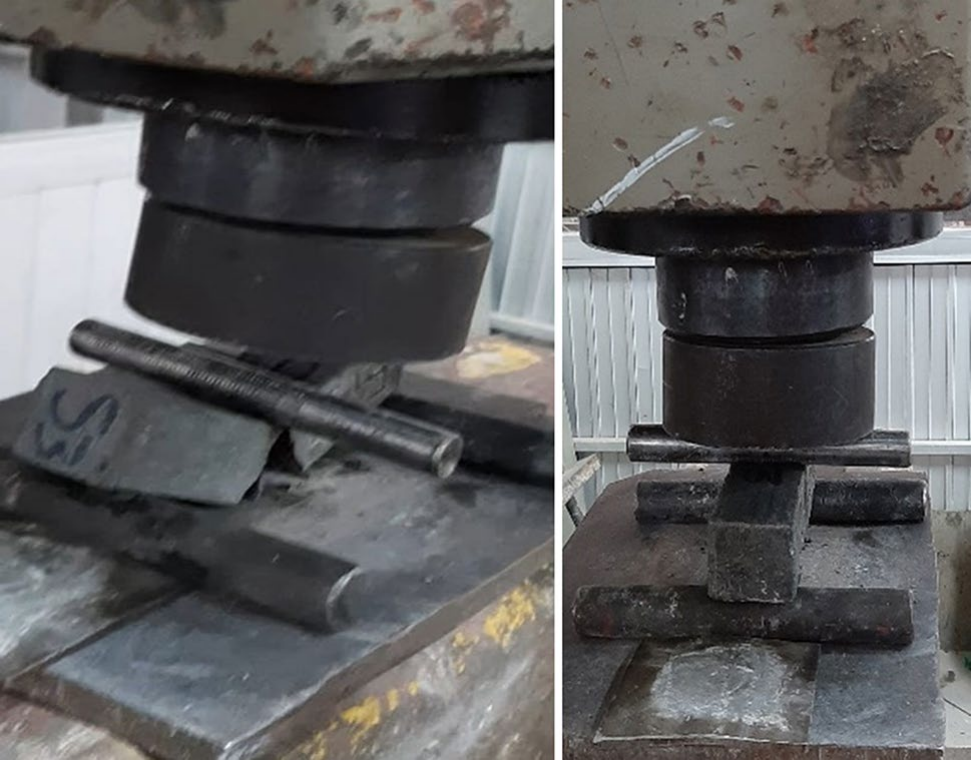
The used electrohydraulic bending and compression testing machine.

Prismatic specimens were implemented to assess the “Flexural Strength” of mortar cubes which were measured by the electrohydraulic bending and compression testing machine (Fig. 13). The “Flexural Strength” of the biocement mortar was found according to Snoeck and De Belie^6^ and Stabnikov et al.^45^, where a prism test was used in which a merely carried prism specimen was inserted by a point load in the prism center. For the flexure strength test, the specimens were cast into 40 * 40 * 160 mm molds and kept in a moist setting at 21 °C for 24 h. Later the molds were separated, and the specimens were cured till test age. Three prism specimens of each treatment were examined after the end of 28 days of curing. The values are expressed in megapascal (MPa). The modulus of rupture (R) of the mortar was determined by the following formula^46,47^:

4 $${\text{Flexural Strength = Pl/ bd}}^{{2}}$$

where: “Flexural Strength in Pa; P = load in N; l = span length between supports in m; b = width of the beam at the point of fracture in m; and h = height of beam at the point of fracture in m”.

### Weights and densities of samples

The densities of mortar cubes were calculated from their weights and dimensions as described by Medvecky et al.^37^.

## Data Availability

All data generated or analyzed during this study are included in this published article.
